# Supplementary material for: Chromosome-level genome sequence of the Genetically Improved Farmed Tilapia (GIFT, Oreochromis niloticus) highlights regions of introgression with O. mossambicus
Source: BMC Genomics. 2022 Dec 15;23:832. doi: 10.1186/s12864-022-09065-8 (PMC9756657; doi:10.1186/s12864-022-09065-8)
Supplement: Supplementary file 2 — Additional file 2: Supplementary Figure 1. Comparison of genomic and genetic positions of the SNP markers of the genetic map. [file 12864_2022_9065_MOESM2_ESM.docx]

**Supplementary Figure 1:** Comparison of genomic and genetic positions of the SNP markers of the genetic map.
