# Supplementary figures and images for: Chromosome-level genome sequence of the Genetically Improved Farmed Tilapia (GIFT, Oreochromis niloticus) highlights regions of introgression with O. mossambicus
Source: BMC Genomics. 2022 Dec 15;23:832. doi: 10.1186/s12864-022-09065-8 (PMC9756657; doi:10.1186/s12864-022-09065-8)

**A**

Topology weighting

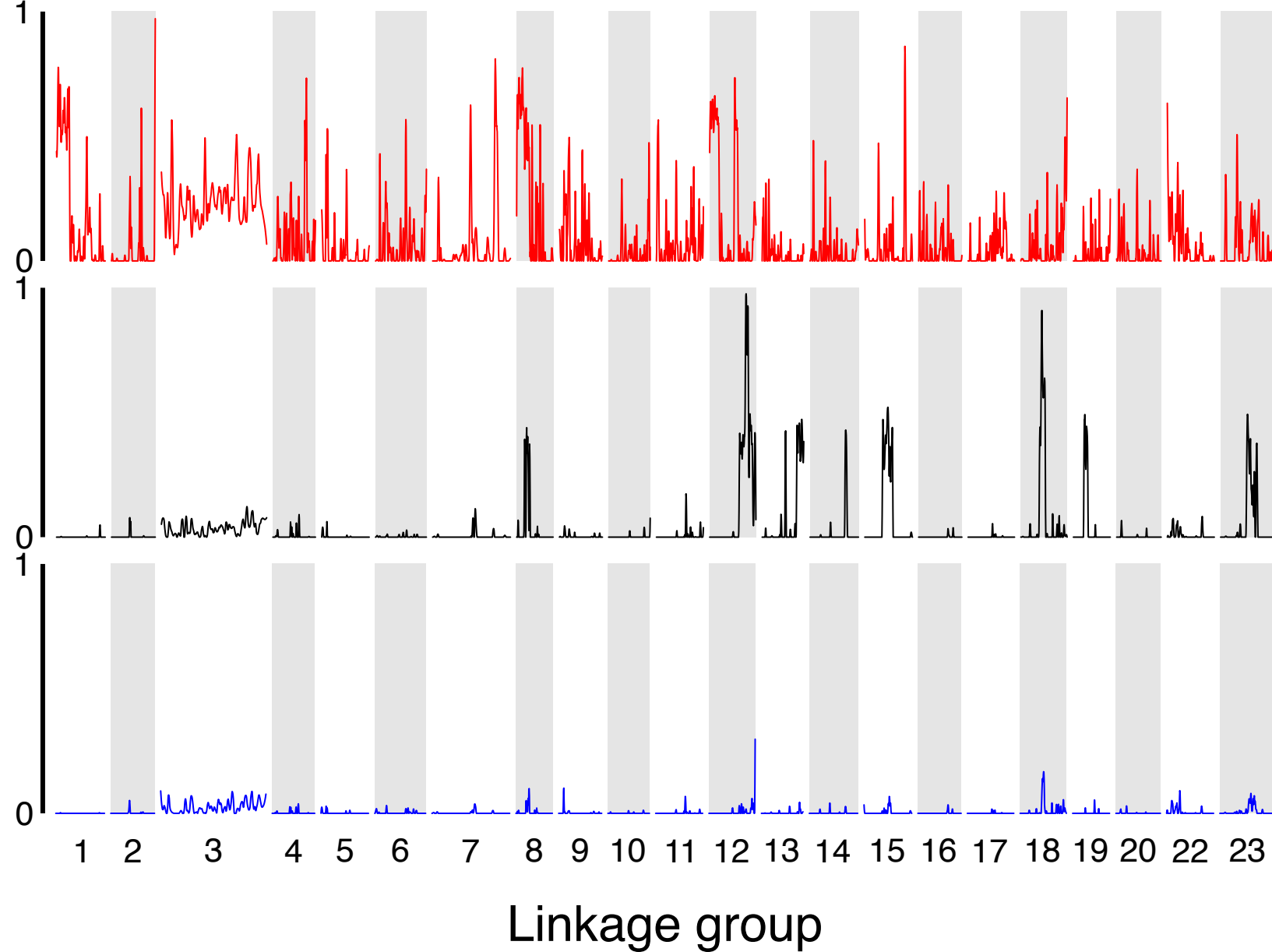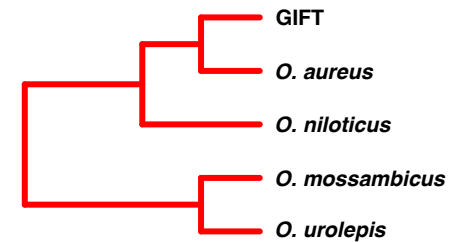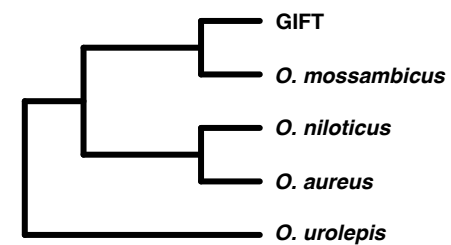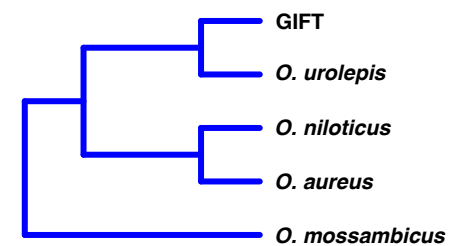**B** Average weighting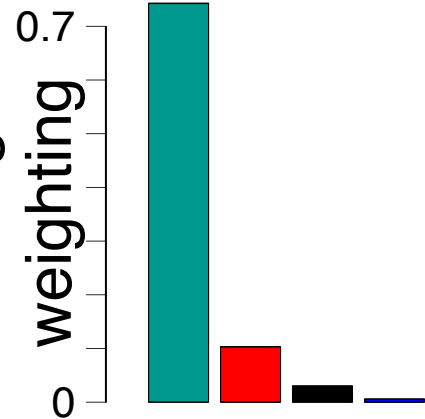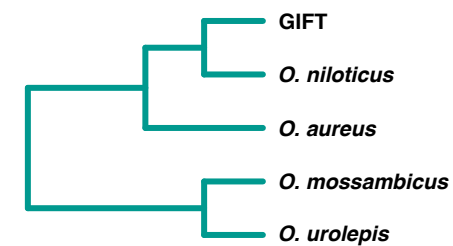

Supplement: Supplementary file 4 — Additional file 4: Supplementary Figure 2. Phylogenetic representation across genomes of species as estimated by TWISST. A) Relative weighting of each phylogeny across the O. niloticus UMD genome assembly. The colours refer to the phylogenies provided in panel B. B) Normalized weights across the O. niloticus UMD genome assembly for the three different phylogenies. [file 12864_2022_9065_MOESM4_ESM.pdf]

**A**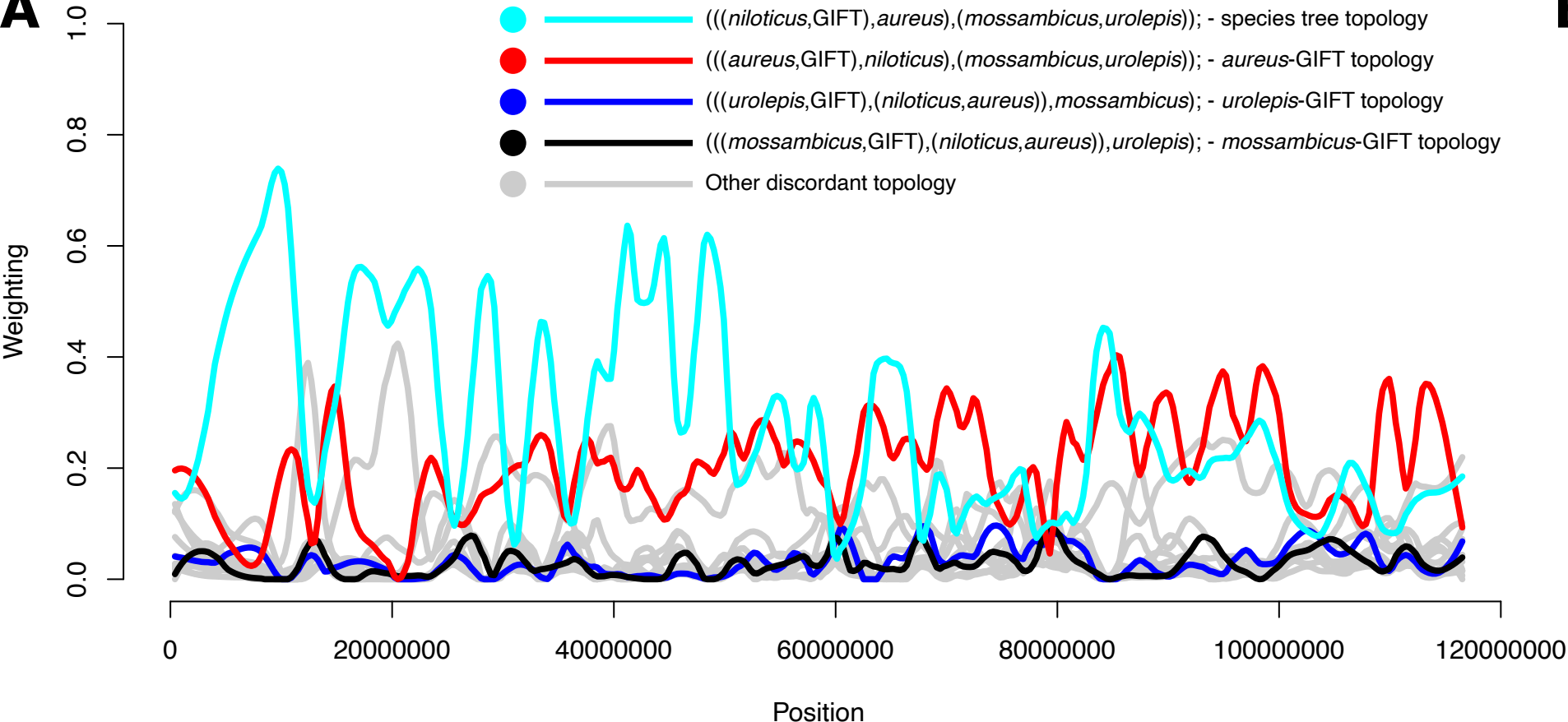**B**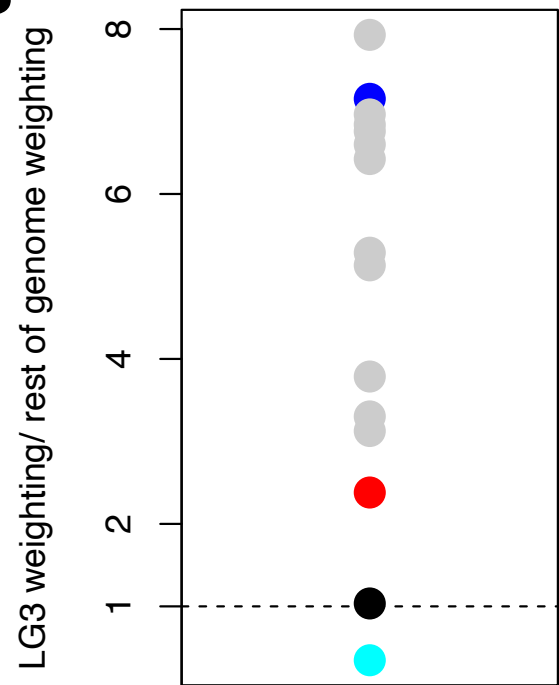

Supplement: Supplementary file 5 — Additional file 5: Supplementary Figure 3. A). Phylogenetic representation across LG3 of species as estimated by TWISST. B) Normalized weights across LG3 for the different phylogenies. [file 12864_2022_9065_MOESM5_ESM.pdf]

A

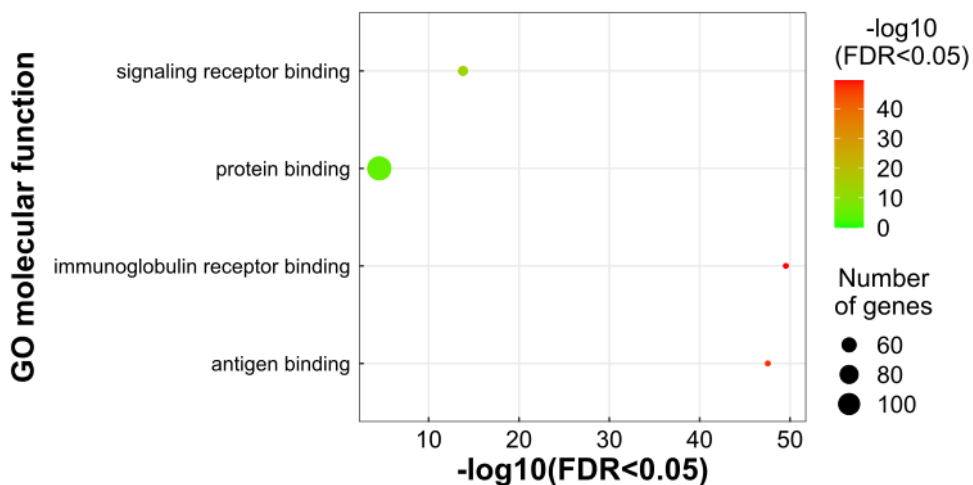

B

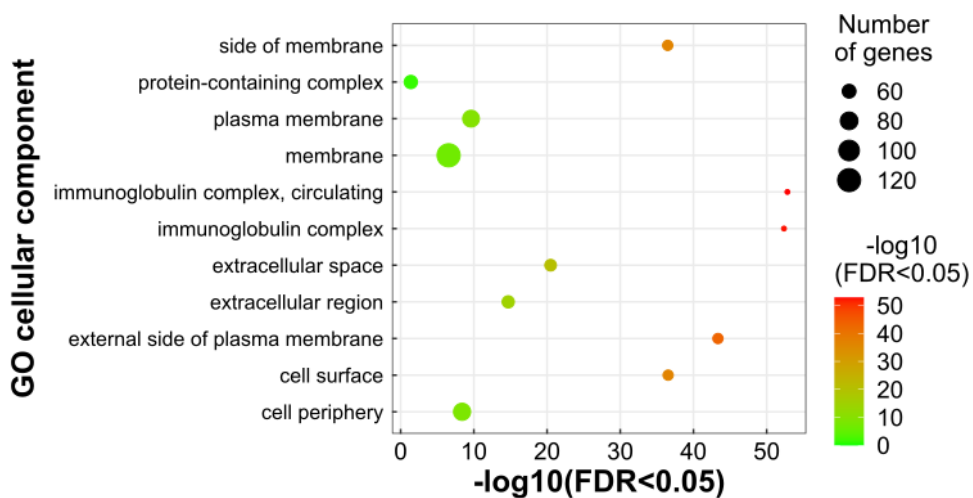

C

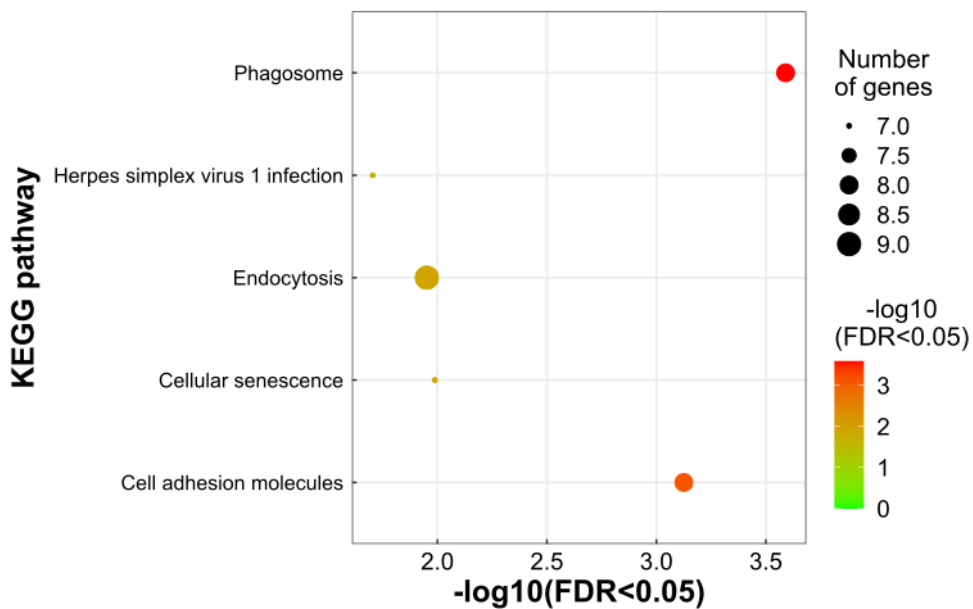

Supplement: Supplementary file 7 — Additional file 7: Supplementary Figure 4. Gene Ontology analysis (Molecular Function, Cellular Components, KEGG Pathway) for the genes identified within O. mossambicus introgressed regions. [file 12864_2022_9065_MOESM7_ESM.pdf]
